# Association between maxillofacial fractures and brain injuries in trauma patients: a cross-sectional study in the Kingdom of Saudi Arabia

**DOI:** 10.11604/pamj.2022.43.193.36283

**Published:** 2022-12-15

**Authors:** Sameer AlGhamdi, Fahad Shaddad Alasmari, Mohammed Bader Alarjani, Hassan Sultan Alamri, Abdullah Ali Aldamkh, Ibrahim Abdullah Alanazi, Musab Bader Alarjani, Abdulrhman Ibrahim Moafa, Nawaf Saad Alrusayyis

**Affiliations:** 1 Department of Family Medicine, College of Medicine, Prince Sattam Bin Abdulaziz University, AlKharj 11942, Saudi Arabia,; 2 Dental Surgery, Armed Forces Medical Services, Riyadh, Saudi Arabia,; 3 Dental Surgery, Advanced Education in General Dentistry Program, Royal Saudi Land forces, Riyadh, Saudi Arabia,; 4 Dentistry, Advanced Education in General Dentistry Program King Salman Armed Forces Hospital, North-western Region, Riyadh, Saudi Arabia,; 5 Dental Surgery, Riyadh Elm University, Riyadh, Saudi Arabia,; 6 Dental Surgery, Armed Forces Medical Services, Airbase Hospital Dhahran, Dhahran, Saudi Arabia,; 7 Royal Land Forces, Ministry of Defense, Riyadh, Saudi Arabia

**Keywords:** Maxillofacial trauma, maxillofacial fractures, traumatic brain injury, Saudi Arabia

## Abstract

**Introduction:**

trauma is on the rise in the Kingdom of Saudi Arabia (KSA) due to rapid urbanization and motorization, posing increased risks of traumatic maxillofacial and brain injuries. Given the high morbidity and mortality associated with these injuries, this study aimed to measure the prevalence and associated factors of brain injury among head injury trauma patients.

**Methods:**

a cross-sectional study was conducted at the King Khalid hospital and Prince Sultan Centre for Healthcare in Al-Kharj City and the Al Kharj Military Industries Corporation Hospital in Al-Kharj City in the KSA. Multivariable logistic regression modelling was performed to ascertain clinical factors associated with Traumatic Brain Injury (TBI).

**Results:**

we included 109 participants aged median 25 and IQR (18-35) years 26.95 ± 14.73 years. Most participants were males (92.7%, n = 101) and 68% (n = 75) had Saudi nationality. About 47.7% (n = 52) had maxillofacial/skull fractures and 44% (n = 48) had TBI. Participants in the age group of 31-40 years experienced a greater risk of TBI than those in the age group of 10 or less years (aOR: 6.2, CI = 1.1p = 0.041). Participants with parietal bone fractures (aOR = 23.1, CI = 3.0 - 181.3, p = 0.003) and frontal bone fractures (aOR = 19.1, CI = 1.7 - 217.0, p = 0.017) were more likely to have TBI compared to those with other skull and facial fractures.

**Conclusion:**

fractures of parietal and frontal bones are associated with a higher risk of TBI in the KSA. Patients with TBI following road accidents with fractures of the frontal or parietal bones, particularly those in the 31-40 age group should therefore be treated with strong suspicion of underlying traumatic brain injury.

## Introduction

Trauma or traumatic injuries contribute to 5.8 million deaths every year, accounting for 10% of all deaths worldwide [1]. Trauma is the fifth leading cause of disability and sixth leading cause of death all over the world [2,3]. Traumatic injuries can affect various parts of the body, resulting in a loss of potential years of productive life with high association of morbidity and mortality in brain and maxillofacial injuries [4,5]. About 69 million people worldwide suffer from traumatic brain injuries (TBI) every year [6]. The prevalence of maxillofacial injuries ranges from 17% to 69% depending on various factors such as the environment, culture, socioeconomic status, and traffic rules/regulations [7]. Concomitant brain and maxillofacial injuries are potentially life-threatening with poor functional and aesthetic outcomes [8].

Maxillofacial fractures are associated with increased morbidity, functional deficit, and longstanding disfigurement requiring multi disciplinary approach [9]. The common causes of maxillofacial fractures are road traffic accidents (RTA), falls, interpersonal violence, and sports [7]. Similarly, TBI are a leading cause of morbidity and mortality, posing a significant health and economic burden worldwide [10]. The major causes of TBI are falls and RTA [11]. A significant association is found between maxillofacial fractures and TBI as a study conducted in Malaysia reported brain injuries in 36.7% of cases of maxillofacial trauma [12]. In addition to functional deficit and aesthetic issues maxillofacial and brain injuries result in a heavy economic burden and poor quality of life [13].

Trauma is on the rise in Saudi Arabia due to rapid urbanization and motorization, posing a significant risk of traumatic maxillofacial and brain injuries in the region [14]. Road traffic accidents account for up to 90.3% of maxillofacial injuries in Saudi Arabia, posing a significant burden of morbidity and mortality due to traumatic injuries [15]. Similarly, RTA are the leading cause of head or brain injuries in Saudi Arabia [16]. Young people are at a high risk of RTA as they are often away from home for education, training, and job [17]. About 40% of Saudi population comprises young people, thus trauma has serious implications on the country´s prosperity as traumatic injuries often result in disabilities and disability adjusted life years (DALYs) [18]. Trauma in young age may therefore require long-term rehabilitative measures, causing an economic burden and low quality of life.

High incidence of RTA in Saudi Arabia points to an increased risk of maxillofacial and brain injuries in the region. Although various studies conducted in Saudi Arabia studied the prevalence and patterns of maxillofacial trauma and head injuries in various circumstances such as RTA, falls, interpersonal violence, or sports, there is lack of studies evaluating the association of maxillofacial fractures and brain injuries in the country. This study was therefore conducted to evaluate the association between maxillofacial fractures and brain injuries among traumatic patients in Saudi Arabia so that further appropriate preventive strategies could be developed to reduce morbidity, mortality, and the economic burden.

## Methods

### Study design and setting

The cross-sectional study was carried out to determine the prevalence and risk factors associated with brain injury among head injury trauma patients managed at two major tertiary care hospitals in the Kingdom of Saudi Arabia (KSA) - The King Khalid hospital and Prince Sultan Centre for Healthcare in Al-Kharj City and the Al Kharj Military Industries Corporation Hospital in Al-Kharj City in the KSA between November 2020 and November 2021.

### Study population

A convenience sampling strategy was used to sample 109 participants from the population of all head injury trauma patients brought to the emergency medicine departments at the study hospitals during the study period. Participants of any age and gender who suffered from a head injury were included in the study. Patients with head injury of nontraumatic etiologies were excluded from the study. Head injury was defined as any major physical injury to the head. Based on the work of Onwuchekwa *et al*. expecting a 32.8% prevalence of parietal fracture among head injury patients [19], a sample of 109 participants was deemed sufficient to achieve 95% confidence and 80% power.

### Data collection

Physicians recorded pertinent patient electronic health information related to age, sex, injury severity scores (ISS), Glasgow Coma Scale (GCS) scores, duration of hospital stay (including stay in the intensive care unit (ICU)), history pertaining to the cause and type of head injury, and radiological imaging reports for detection and assessment of maxillofacial fractures and traumatic brain injuries.

### Definitions

Presence of traumatic brain injury was selected as the outcome variable. Age, gender, cause of injury, type of head injury (penetrating vs non-penetrating), admission to the intensive care unit (ICU) and presence and type of skull fracture sustained were the explanatory variables studied.

### Statistical analysis

The data from our study were tabulated and analyzed using IBM´s statistical software - Statistical Package for Social Services version 20 (Armonk, United States). We computed descriptive statistics for all variables in the study. We summarized quantitative variables with mean and standard deviation. Frequencies and percentages were calculated for categorical variables. There were no missing data for any variables of interest. We used logistic regression to assess the association between the relevant patient characteristics and traumatic brain injury. We conducted a univariable regression for each potential risk factor. The variables that showed evidence of association with TBI at p < 0.25 were entered into a multivariable binary logistic regression. Odd ratios were calculated to achieve 95% confidence with the threshold for type 1 error rate set to p = 0.05.

### Ethical considerations

Our team obtained ethical clearance from the Medical Ethics Committee of the Prince Sattam Bin Abdulaziz University before beginning data collection. (Ethics approval RUH-RDF-54924) Written and informed consent was sought from the study participants or their legal guardians where applicable (in case of children and those whose injuries precluded them from giving informed consent) before they were enrolled in the study. All 109 patients who were eligible to participate in the study consented to do so. All obtained patient data were completely anonymized to protect the participants´ privacy.

## Results

### General characteristics of the study population

We included 109 participants with a median age of 25 and IQR (18-35) years. Most of the participants were male (92.7%, n = 101) and 68.8% (n = 75) had Saudi nationality. Mean participant ISS and GCS calculated at the time of admission to the hospital were 21.6(12.65) and 13.03(2.85). Most head injuries were sustained following road traffic accidents (RTA), 97 (89%), specifically those involving cars, 92 (84.4%). Nearly all - 98.2% (n = 107)- head injuries were not penetrating with almost half - 47.7% (n = 52) - having maxillofacial fractures. Forty-four percent (44%) (n = 48) trauma participants had brain injuries ascertained by radiological imaging of the head. On average patients required 10 (18.6) days of hospitalization with most - 82.6%, (n = 90) - recovering without needing ICU admissions. Participant characteristics are reported in Table 1.

**Table 1:** demographic and clinical characteristics of the study subjects

Study variables		Frequency	Percentage (%)
Age group	10 years or less	17	15.6
	11-20 years	21	19.3
	21-30 years	37	33.9
	31-40 years	18	16.5
	More than 40 years	16	14.7
Sex	Female	8	7.3
	Male	101	92.7
Nationality	Non-Saudi	34	31.2
	Saudi	75	68.8
Type of accident	Industrial	12	11.0
	RTA	97	89.0
Cause	Car	92	84.4
	Fall from height	12	11.0
	Hit by car	2	1.8
	Motor bike	3	2.8
Type of head injury	Non-Penetrating	107	98.2
	Penetrating	2	1.8
Skull Fracture	Yes	52	47.7
	No. of Fractures 1/2/3/4	33/10/5/3	30.3/9.2/4.6/2.8
Brain Injury	Yes	48	44.0
	No. of Injuries 1/2/3/4	35/8/3/1	32.1/7.3/3.7/0.9
ICU Admission	Yes	19	17.4
	No	90	82.6

### Patterns of craniofacial fractures and brain injuries

Parietal (13.8%, n = 15), temporal (10.1%, n = 11), and frontal (7.3%, n = 8) bone fractures were the three most frequently seen craniofacial fractures in our study population. Epidural hematoma or hemorrhage (15.6%, n = 17), subarachnoid hemorrhage (10.1%, n = 11), subdural hematoma or hemorrhage (10.1%, n. = 11), and cerebral contusions (7.3%, n = 8) were the most seen patterns of traumatic brain injury in the population. All patterns of maxillofacial fractures and brain injuries seen in our population are tabulated in Table 2.

**Table 2:** patterns of maxillofacial fractures and brain injuries

Maxillofacial/Skull Fractures	n (%)	Brain Injuries	n (%)
Parietal	15(13.8%)	Epidural Hematoma/Hemorrhage	17(15.6%)
Temporal	11(10.1%)	Subarachnoid Hemorrhage	11(10.1%)
Frontal	8(7.3%)	Subdural Hematoma/Hemorrhage	11(10.1%)
Maxilla	6(5.5%)	Cerebral Contusion	8(7.3%)
Nasal	6(5.5%)	Brain/Cerebral edema	8(7.3%)
Orbital	6(5.5%)	Subgaleal hematoma	5(4.6%)
Mandible	6(5.5%)	Eye injury	3(2.8%)
Occipital	5(4.6%)	Intra-ventricular Hematoma	3(2.8%)
Zygomatic arch	4(3.7%)	Ecchymosis	2(1.8%)
Facial	3(2.8%)	Other (Pneumocephalus, Sub-capsular hematoma, Palate injury etc.)	7 (6.4%)
Maxillary sinus	2(1.8%)		
Other (Base, Sphenoid, Bicondylar, Para-symphysis etc.)	5(4.6%)		

### Clinical factors associated with traumatic brain injuries

Univariable analysis of different clinical factors and the presence or absence of traumatic brain injury is provided in Table 3. Covariates that showed strong evidence of an association with the outcome (p < 0.25) were considered for multivariable regression modelling. The final multivariable logistic regression model included covariates age and the different patterns of maxillofacial fractures (Table 3). Only three of the included covariates appeared statistically significant. In our univariate analysis having a fracture of the parietal (OR =11.0, CI = 2.3- 51.4, p = 0.002), temporal (OR = 6.8, CI = 1.4 - 33.2, p = 0.018), or frontal bone (OR = 10.2, CI = 1.2 - 86.4, p = 0.032) were significantly associated with traumatic brain injury. Age, fracture of the parietal or frontal bones had a significant association with traumatic brain injury after adjusting for potential confounders in the multivariable analysis as detailed below.

**Table 3:** association of brain injuries with demographic, clinical characteristics, and type of maxillofacial/skull fracture

Factors		Brain Injury/ Injuries		Univariate Analysis		Multivariate Analysis	
		Yes	No	OR (95%CI)	Sig.	OR (95%CI)	Sig.
Age group	10 years or less	4(23.5%)	13(76.5%)	1	-	1	0.159
	11-20 years	11(52.4%)	10(47.6%)	3.57(0.87 -14.64)	0.077*	4.3(0.77-24.15)	0.097
	21-30 years	18(48.6%)	19(51.4%)	3.07(0.84 -11.21)	0.088*	3.64(0.76-17.42)	0.106
	31-40 years	9(50%)	9(50%)	3.25(0.76-13.88)	0.112*	6.2(1.07-35.74)	0.041*
	> 40 years	6(37.5%)	10(62.5%)	1.95(0.43-8.82)	0.386	0.98(0.13-7.17)	0.986
Sex	Female	4(50%)	4(50%)	1	-	-	-
	Male	44(43.6%)	57(56.4%)	0.77(0.18-3.26)	0.725	-	-
Type of Accident	Industrial	5(41.7%)	7(58.3%)	1	-	-	-
	RTA	43(44.3%)	54(55.7%)	1.11(0.33-3.75)	0.861	-	-
Cause	Car	41(44.6%)	51(55.4%)	1	-	-	-
	Fall from height	5(41.7%)	7(58.3%)	1.12(0.33-3.80)	0.849	-	-
	Hit by car	1(50%)	1(50%)	1.40(0.07-28.1)	0.826	-	-
	Motorbike	1(33.3%)	2(66.7%)	0.70(0.04-10.01)	0.793	-	-
Type of Head Injury	No	46(43%)	61(57%)	N/a	N/a	-	-
	Yes	2(100%)	0(0%)	N/a	N/a	-	-
Skull Fracture	No	21(36.8%)	36(63.2%)	1	-	-	-
	Yes	27(51.9%)	25(48.1%)	1.85(0.86-3.98)	0.115*	0.32(0.09-1.21)	0.093
Parietal	No	35(37.2%)	59(62.8%)	1		-	
	Yes	13(86.7%)	2(13.3%)	10.95(2.33-51.4)	0.002*	23.11(2.95-181.3)	0.003*
Temporal	No	39(39.8%)	59(60.2%)	1		-	-
	Yes	9(81.8%)	2(18.2%)	6.80(1.39-33.2)	0.018*	3.86(0.47-31.8)	0.209
Maxilla	No	46(44.7%)	57(55.3%)	1	-	-	-
	Yes	2(33.3%)	4(66.7%)	0.62(0.10-3.53)	0.59	-	-
Nasal	No	46(44.7%)	57(55.3%)	1	-	-	-
	Yes	2(33.3%)	4(66.7%)	0.62(0.10-3.53)	0.59	-	-
Orbital	No	43(41.7%)	60(58.3%)	1	-	-	-
	Yes	5(83.3%)	1(16.7%)	6.97(0.78-61.8)	0.081*	5.41(0.37-78.64)	0.216
Mandible	No	47(45.6%)	56(54.4%)	1	**-**	-	-
	Yes	1(16.7%)	5(83.3%)	0.238(0.07-2.11)	0.198*	0.84(0.07-9.96)	0.887
Occipital	No	46(44.2%)	58(55.8%)	1	-	-	-
	Yes	2(40%)	3(60%)	0.84(0.13-5.24)	0.852	-	-
Zygomatic arch	No	45(42.9%)	60(57.1%)	1	-	-	-
	Yes	3(75%)	1(25%)	4.0(0.40-39.73)	0.237*	5.55(0.35-88.57)	0.225
Facial	No	48(45.3%)	58(54.7%)	N/a	N/a	-	-
	Yes	0(0%)	3(100%)	N/a	N/a	-	-
Maxillary sinus	No	46(43%)	61(57%)	N/a	N/a	-	-
	Yes	2(100%)	0(0%)	N/a	N/a	-	-
Frontal	No	41(40.6%)	60(59.4%)	1	**-**	-	-
	Yes	7(87.5%)	1(12.5%)	10.2(1.21-86.4)	0.032*	19.1(1.68-216.9)	0.017*

For Uni-variate analysis significance level set at <0.25; For Multivariate analysis significance level set at <0.05; OR: Odds ratio; CI: Confidence interval; *: Significant

**Age:** half of the (50% n = 9) participants of 31-40 years of age had traumatic brain injury compared to 23.5% (n = 4) of 10 or less years of age. Patients in the 31-40 years group were found to be 6.2-times more likely to have traumatic brain injuries compared to the 10 or less years group, 6.20 (1.07 - 35.74), *p*= 0.041.

### Parietal bone fractures

A majority (86.7%, n = 13) of participants with parietal bone fractures had traumatic brain injury compared to 37.2% (n = 35) participants without parietal bone fractures. Participants with parietal bone fractures were 23.11-times more likely to have traumatic brain injury than those without parietal bone fractures (aOR =23.11, CI: 2.95 - 181.29, *p*= 0.003).

### Frontal bone fractures

Most of the (87.5%, n = 7) participants with frontal bone fractures had traumatic brain injury as against 40.6% (n = 41) participants without frontal bone fractures. Participants with frontal bone fractures were 19.10-times more likely to have traumatic brain injury than those without frontal bone fractures (aOR= 19.1, CI = 1.68 - 217.0, *p*= 0.017).

## Discussion

In this study, we set out to find the prevalence and risk factors associated with brain injury among head injury trauma patients. We found that having fractures of frontal or parietal bones was associated with a significant increase in the odds of brain injury. Though fractures of the orbital bone and zygomatic arch were associated with an increased risk of brain injury, these associations did not meet the threshold of statistical significance. Additionally, men in the age group of 31 - 40 had the highest risk of brain injury. In our study, as in several studies conducted in Saudi Arabia, the middle east, and around the world [20-22] most head injuries were caused by car accidents, emphasizing the need for improving road safety measures.

### Associations between brain injury and craniofacial fractures

Our study found a strong association between brain injury and fractures of the frontal and parietal bone. This concurs with the findings of Aldwsari *et al*. [23] conducted at the King Khalid Hospital and Prince Sultan Centre for Health Services in the KSA in 2018. In addition to fractures of the frontal and parietal bones, Aldwsari *et al*. [23] found fractures of the temporal and occipital bones to be significantly associated with brain injury. Similarly, a study conducted by Abosadegh *et al*. [24] in Malaysia found that brain injury was most associated with cranial bone fractures. These findings highlight the importance of investigating brain injury among patients with skull fractures and vice versa.

Our study found an association, albeit not statistically significant, between brain injury and fractures of the zygomatic arch and orbital bones. This association was also reported by Aldwsari *et al*. [23] who found strong evidence of association between orbital fractures and brain injury and weak evidence of association between fractures of the zygomatic arch and brain injury. Aldwsari *et al*. also found strong evidence of an association between traumatic brain injury and fractures of the maxillary sinus and nasal bone. A similar study from Malaysia [24] found the zygomatic arch, orbital wall, maxillary sinus wall, and alveolar processes of mandible fractures to be significantly associated with brain injury. This suggests that those with orbital, zygomatic arch, or other maxillofacial fractures should be regarded with a strong supposition of underlying brain injury. Though we did not grade the severity of facial fractures in this study, You *et al*. [25] found that the severity of facial fractures was correlated to the severity of traumatic brain injury. Further study is required to determine the correlation between the severity of facial fractures and the severity of brain injury in the Saudi population. Concurrent injuries to skull base and cervical spine are also important considerations in patients with maxillofacial or cranial fractures [5] and merit further study.

This study was conducted in three major tertiary care hospitals in the Kingdom of Saudi Arabia (KSA) in Al-Kharj City. This may not be generalizable to the population of Saudi Arabia or other neighboring countries. This project studied only patients who reached the emergency department alive and thus could not measure the association between maxillofacial fractures and brain injuries among participants who died before reaching the hospital. The results of our study may therefore have been influenced by a survivor bias as patients with serious head injuries may have died before reaching the hospital.

## Conclusion

Our study found a significant association between brain injury and fractures of the frontal and parietal bones among patients from the Kingdom of Saudi Arabia. The study found traumatic brain injury to be associated with fractures of the orbit and zygomatic arch. However, we did not find strong evidence of these results as the association was not statistically significant.

### What is known about this topic

- Trauma or traumatic injuries contribute to 5.8 million deaths every year, accounting for 10% of all deaths worldwide;
- Trauma is the fifth leading cause of disability and the sixth leading cause of death all over the world;
- About 69 million people suffer from traumatic brain injuries every year.

### What this study adds

- Parietal, temporal, and frontal bone fractures are the most frequent traumatic craniofacial fractures in Saudi Arabia;
- Fractures of parietal and frontal bones are associated with an increased risk of traumatic brain injury in Saudi Arabia;
- Fractures of the orbital bone and zygomatic arch are also associated with an increased risk of brain injury, although these associations are not statistically significant.
